# *Cryptosporidium* Oocysts in a Water Supply Associated with a Cryptosporidiosis Outbreak

**DOI:** 10.3201/eid0806.010271

**Published:** 2002-06

**Authors:** Andrew D. Howe, Sue Forster, Stephen Morton, Roberta Marshall, Keith S. Osborn, Peter Wright, Paul R. Hunter

**Affiliations:** * East Lancashire Health Authority, Nelson, United Kingdom; † United Utilities Water PLC, Warrington, United Kingdom; ‡ Public Health Laboratory, Preston, United Kingdom; § University of East Anglia, Norwich, United Kingdom

**Keywords:** *Cryptosporidium Oocysts* in a Water Supply Associated with a Cryptosporidiosis Outbreak, Cryptosporidium, outbreak, oocysts, water, zoonosis, biofilm

## Abstract

An outbreak of cryptosporidiosis occurred in and around Clitheroe, Lancashire, in northwest England, during March 2000. Fifty-eight cases of diarrhea with *Cryptosporidium* identified in stool specimens were reported. *Cryptosporidium* oocysts were identified in samples from the water treatment works as well as domestic taps. Descriptive epidemiology suggested that drinking unboiled tap water in a single water zone was the common factor linking cases. Environmental investigation suggested that contamination with animal feces was the likely source of the outbreak. This outbreak was unusual in that hydrodynamic modeling was used to give a good estimate of the peak oocyst count at the time of the contamination incident. The oocysts’ persistence in the water distribution system after switching to another water source was also unusual. This persistence may have been due to oocysts being entrapped within biofilm. Despite the continued presence of oocysts, epidemiologic evidence suggested that no one became ill after the water source was changed.

Outbreaks of cryptosporidiosis associated with drinking water have been an emerging problem for the past 20 years. In the 1990s, cryptosporidiosis became the most common cause of outbreaks associated with public drinking water supplies in the United Kingdom (1). This disease is also responsible for several of the largest outbreaks of waterborne disease seen in the United States (1). Yet substantial areas of uncertainty over many aspects of the epidemiology of this infection remain. One of the most pressing such areas is determining what concentration of oocysts in drinking water is considered safe.

In the United Kingdom, recent legislation was enacted that set a legal limit of 1 oocyst/ 10 L when water was sampled continuously over a 24-hour period (2). However, this level was set as a treatment standard and was not derived from known public health standards. With current knowledge, proposing standards for cryptosporidia based on public health criteria is not possible, primarily because published reports of outbreaks have not had accurate measures of the concentration of oocysts in the water at the time when infection was thought to have occurred. We report, to our knowledge, the first outbreak to have occurred when a fairly accurate estimate of the concentration of oocysts in the water could be made.

## The Outbreak

In March 2000, an outbreak of cryptosporidiosis occurred in and around the town of Clitheroe in Lancashire County in northwest England. This small market town, nestled in the hills near the Ribble River, is a thriving community that attracts many tourists. The surrounding countryside supports arable and dairy farming. Before this outbreak, reported cases of cryptosporidiosis were low. In the years 1997–1999, the mean annual attack rate of laboratory-confirmed cryptosporidiosis was 4.83 per 10,000 residents per year, compared with 13.57 for the region as a whole.

During March 1–15, 2000, the Ribble Valley Environmental Health Department reported nine cases of cryptosporidiosis to the East Lancashire Health Authority. All the patients lived in or near Clitheroe. Provisional information provided by the water company indicated that six of these nine patients lived in a single water zone supplied by the same water treatment works. On the basis of this information, an outbreak was declared, and an outbreak control team was established. The team met for the first time on March 16.

## Methods

### Epidemiologic Investigation

Environmental health and public health department personnel interviewed patients with cryptosporidiosis in person or by telephone, using a structured questionnaire (3). Analysis was performed by using the computer program Epi-Info (version 6.02; Centers for Disease Control, Atlanta, GA). Patients were defined as those with a positive stool sample who lived in or visited the implicated water zone and who had onset of diarrhea since March 1, 2000. Cases were defined as primary when no other member of the household had had diarrhea in the 2 weeks before the onset of symptoms; possible secondary cases were defined as those in which a member of the same household had had diarrhea in the previous 2 weeks. The case definitions included those who had traveled abroad for <7 days.

### Microbiologic Investigation

General practitioners in the area submitted stool samples to the local hospital microbiology laboratory. Stools were examined by microscopy with the modified auramine phenol stain (4). Positive samples were then sent to the Public Health Laboratory Service’s Cryptosporidium Reference Unit for genotyping.

### Environmental Investigations

The local water company provided information on the water supply, instituted a water-sampling schedule (from domestic properties, water treatment works, and fire hydrants during flushing operations), and analyzed the water samples to identify *Cryptosporidium* oocysts. Most of the samples were 10-L grab samples analyzed according to the U.K. standard method (5). The large-volume samples were analyzed by the method in the Water Supply (Water Quality) Amendment Regulations of 1999 (2). The source of water to the affected area (Grindleton Springs) was visited by members of the outbreak control team.

The local water company supplied rainfall statistics for the weeks preceding the outbreak. Local authority engineers were consulted for information on previous high water or flood warnings.

After the incident, the water company constructed a physical model of the affected reservoir, Lowcocks, with a geometric scaling ratio of 32:1. Flows were tracked by using salt injection with an array of conductivity probes suspended above the tank and injecting colored dyes for visualization. As the ratio of the two respective inlet flows can vary, the baseline performance of the tank was evaluated over a range of operational, but steady state, conditions. A series of transient tests was then conducted to mirror the operation of the reservoir in the time leading up to and covering the incident until the boil water notice was issued on March 21.

## Results

### Descriptive Epidemiology

**Figure 1:**
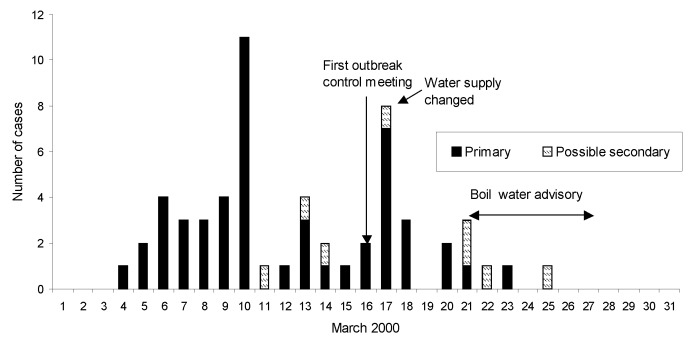
Date of diarrhea onset, 58 cryptosporidiosis cases, Clitheroe, 2000.

Fifty-eight cases met the case definition. Of these, three were in patients who had traveled abroad for <7 days in the 2 weeks before illness. Fifty-one cases were identified as primary, and seven as possible secondary. The dates of onset of cases (Figure 1) showed peaks on March 10 and 17. Ages of patients ranged from 7 months to 95 years, but most patients were <5 years (52%). Thirty (52%) of the patients were male and 28 (48%) female. All 58 patients (100%) had diarrhea; 18 (31%) had fever, 48 (83%) abdominal pain, 19 (33%) vomiting, and three (5%) blood in the stool.

Fifty-one patients lived in the same water supply zone and drank unboiled main tap water in the zone. The crude attack rate for residents of this zone was 29.6 per 10,000 population (based on general practitioner registered population of 17,252 linked by postal code of residences in the water supply zone). The crude attack rate for people within the same local government area but not living in the same water supply zone was 1.8 per 10,000 population, giving a relative risk associated with residence in the implicated water supply zone of 16.2 (95% confidence interval 7.5 to 35.0). The age-specific attack rate varied from 275 per 10,000 in children <5 years of age to 5.6 per 10,000 in those >44 years (Table 1). Seven patients lived in properties not in the affected water zone. However, six of these had drunk unboiled main water in the affected zone in the 2 weeks before illness; the other patient had visited a swimming pool in the zone. Other potential risk factors, such as travel, visit to a swimming pool, and consumption of certain foods, were included in the questionnaire. None was common in patients.

**Table 1:** Age-specific attack rates for cryptosporidiosis in residents of water zone 97, Clitheroe, March 2000

Age group	Cases (n=51)	Population	Rate/10,000	95% confidence interval
<4	26	945	275.1	170.8 to 379.4
5–14	9	2,283	39.4	13.7 to 65.1
15–44	12	6,822	17.6	7.6 to 27.5
>45	4	7,202	5.6	0.1 to 11.0
Total	51	1,7252	29.6	21.5 to 37.7

### Microbiologic Testing

Of the 58 cases with a positive stool sample for *Cryptosporidium,* 47 specimens were typed. All were *C. parvum* genotype 2 (for nine cases there was insufficient material, and two specimens were untypable).

### Environmental Results

#### Water Sample Analysis

Lowcocks Water Treatment Works (WTW), sourced from Grindleton Springs, supplied approximately 90% of the water to the affected zone. The supply was a spring source that fed a single service reservoir and from there moved into distribution. However, the reservoir could also be filled from a nearby larger water supply via an aqueduct. The supply was chlorinated but not filtered. As part of the risk assessment carried out under water quality amendment regulations (2), Lowcocks WTW was classified as being at “significant risk” from *Cryptosporidium* oocysts in water supplied from the works. However, continuous monitoring had not yet begun before the outbreak.

The reservoir is rectangular with two inlets and a single outlet. The tank is 110 m long and 90 m wide with an operational depth between 3.5 m and 5.4 m. The spring has one inlet, which varies from 2 to 6 megaliters per day and another from the aqueduct, which varies from 1.5 to 5 megaliters per day. The calculated capacity of the reservoir is 53 megaliters. The ratio of aqueduct to spring water varies considerably during normal operation; full advantage is taken of the increase in availability of the spring’s source after major rainfalls.

**Figure 2:**
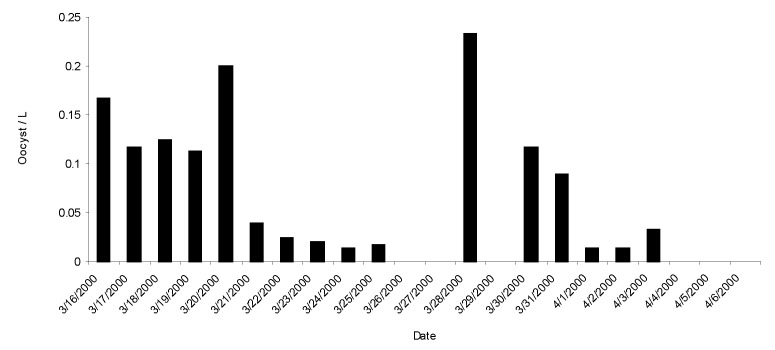
Mean daily cryptosporidia oocyst counts from 10-L grab samples taken during outbreak

On March 17, a large-volume sample of water (1,627 L) from a pumping station fed from Lowcocks WTW yielded 76 oocysts of *Cryptosporidium* per 1,000 L. *Cryptosporidium* oocysts were also identified in a water sample taken from a domestic tap in the water zone on March 16 at a concentration of five oocysts per 10 L of water. From March 16 to April 6, a total of 192 samples (10-L grab samples) from domestic taps or fire hydrants in the affected zone were analyzed; 47 (24%) contained *Cryptosporidium* oocysts in concentrations ranging from 1 to 9/10 L. Six water samples from domestic taps in areas adjoining the affected water zone were negative (Table 2, Figure 2).

**Table 2:** Results of 10-L grab samples taken within distribution range of water works during investigation^a^ of cryptosporidial outbreak, Clitheroe, March 16–April 6, 2001

Date	Samples taken	Samples positive	Oocyst counts of positive samples/L				
16 Mar	3	1	0.5				
17 Mar	6	5	0.1	0.2	0.1	0.2	0.1
18 Mar	8	4	0.2	0.2	0.3	0.3	
19 Mar	8	5	0.2	0.3	0.1	0.1	0.2
20 Mar	9	5	0.1	0.2	0.9	0.5	0.1
21 Mar	23	5	0.2	0.1	0.1	0.4	0.1
22 Mar	16	4	0.1	0.1	0.1	0.1	
23 Mar	15	2	0.1	0.2			
24 Mar	15	2	0.1	0.1			
25 Mar	12	2	0.1	0.1			
26 Mar	12	0					
27 Mar	9	0					
28 Mar	3	2	0.3	0.4			
29 Mar	3	0					
30 Mar	6	3	0.1	0.2	0.4		
31 Mar	9	3	0.1	0.1	0.6		
1 Apr	7	1	0.1				
2 Apr	7	1	0.1				
3 Apr	6	2	0.1	0.1			
4 Apr	6	0					
5 Apr	6	0					
6 Apr	3	0					
^a^ Total volume examined each day (in L) = 10 X number of samples taken.							

### Site Visits

The concrete casings of two of the Grindleton Springs collection chambers showed signs of aging and were in a poor state of repair (one could look directly into one chamber through holes in the concrete). Evidence of recent livestock excreta (cattle) was present in the areas around, and in direct contact with, the covers to several of the spring collection chambers; manure was also spread in a field within 5 m of one wellhead.

#### Rainfall Statistics

Abnormally heavy rainfall (up to 58 mm per day) and flood alerts were reported for the area on February 27 and March 2–7.

#### Hydraulic Modeling

A number of detailed transient state tests were conducted in which the flows and levels were altered in line with the reservoir operation before and during the outbreak. Initially, the first “injection” of oocysts was assumed to have come into the reservoir on February 27, after the first associated heavy rainfall. However, results from these initial tests indicated that, because of the way the reservoir operated and its short nominal retention time (2 days) during part of this period, a large spike of oocysts entering the reservoir from the springs inlet on February 27 would have been effectively washed out by the time the sample was taken on March 17.

Two potential contamination events, one after each major rainfall event on February 27 and March 2, respectively, were then proposed. This hypothesis was modeled by injection of two discrete salt pulses into the model springs inlet at the appropriately scaled time in the modeling run. Results indicated three peaks of oocyst counts at the tank outlet. The first peak occurred when the tank was operating on only spring flow, corresponding to February 29. The second peak came on March 1, when aqueduct flow was introduced. The final peak occurred on March 2–3, after the second salt pulse (simulating the rainfall incident).

Based on the concentration found in the March 17 sample, the most probable peak concentration that the Clitheroe population would have been exposed to was 40 times greater, approximately 30 oocysts per 10 L. These values are based on tests in which the pulse was introduced instantaneously; in practice, contamination likely took place over several hours or days after each major rainfall event. While it is likely that the behavior of oocysts would not substantially differ in the water system and the salt and dye model, these numbers should not be considered exact; rather, they are a good indication of level of exposure over the period in question.

#### Control Measures

At the first outbreak control team meeting, 11 of 14 reported cryptosporidiosis cases were known to be in residents of the same water supply zone. As a result, the water supply to the affected area was changed to an alternate supply during the following night, and the system was flushed. The alternate supply was an approximately 50/50 blend of filtered surface water from two separate (protected) upland impounding reservoirs. The first source (Watchgate) provides up to 600 megaliters per day to a population of approximately 1.75 x 10^6^; the second source (Hodder) provides up to 50 megaliters per day to a population of approximately 1.75 x 10^3^. Both areas had had no observed increase in the rates of reported cryptosporidiosis.

At the third outbreak control team meeting, when results of sampling became available, it became evident that, although the water supply to the area had been changed by 9:30 a.m. on March 17 (and its distribution throughout the zone confirmed by chemical analysis of domestic water samples), substantial numbers of *Cryptosporidium* oocysts still existed in samples taken during the next 4 days (March 17–20). Initial samples from the source of the new water supply showed no evidence of contamination. Historic archived data available for both new sources showed only a low frequency of detected oocysts in the raw (untreated source) water for each site. During the incident, five samples of treated water were taken from the first site and 13 samples from the second source. A single oocyst was reported in one 10-L sample taken from the first site; no oocysts were detected in the other samples.

The outbreak control team agreed that there continued to be a risk to public health and issued a “Boil Water Advisory” on March 21. This advisory was rescinded on March 27 after extensive water system flushing operations and 2 days of domestic water samples being clear of *Cryptosporidium* oocysts. The peak in counts on March 28, although calculated from three samples, was associated with the sampling water from hydrants rather than from domestic taps.

Water sampling continued, but samples were taken from fire hydrants rather than domestic taps. While inspections of the water system showed no evidence of ongoing contamination, analysis of water continued to show cryptosporidia. When oocysts were detected in hydrant samples after the source of water had been changed, experienced operations staff inspected the route of the aqueduct, and boundary valves at the periphery of the affected distribution system were checked to ensure that water could not enter this system from an adjacent zone.

At this stage, no further new cases of cryptosporidiosis were being reported. The original source of water, Grindleton Springs, had been identified as having a plausible source of oocysts within the watershed (cattle excreta), a plausible pathway (through the damaged spring head structure to one of the chambers), and inadequate treatment for removing oocysts (microfiltration with a pore size >40 µ); this source of water had been isolated and discharged to waste. Thus, the change in sampling method, rather than ongoing contamination, might be causing the continuing positive oocyst results. For this reason, the boil water advisory was not reinstituted. Further flushing continued, no new cases of cryptosporidiosis were reported, and the last water sample positive for oocysts was on April 3.

## Discussion

Use of U.K. Public Health Laboratory Service guidelines strongly associated this outbreak with the water supply because *Cryptosporidium* oocysts were detected in treated water and the descriptive epidemiology suggested that drinking tap water was the only common factor linking the cases (6). Environmental investigations suggested that contamination of Grindleton Springs with animal feces was the probable cause of the outbreak. Results of genotyping were consistent with an animal source.

This outbreak is unusual because of the very high attack rate of laboratory-confirmed cases. The crude attack rate for microbiologically confirmed cases of cryptosporidiosis was much higher than previously reported in the United Kingdom (7–9). We suggest that this high attack rate occurred because of low immunity in the population and the probable high concentration of oocysts at the time of the initial contamination. Although we have no direct measure of population immunity before this outbreak, the incidence of infection in previous years was low compared with that in the rest of the region. Furthermore, until the outbreak, the water supply was a groundwater source; various groups have suggested that such sources are associated with lower sporadic infections and lower population immunity (7,10).

The other major issue raised by this outbreak was the impact of changing the source of water. The outbreak control team had suggested that changing the water supply to the affected area at the beginning of the outbreak would remove the *Cryptosporidium* oocysts from the water. However, this measure did not result in the expected immediate clearance of contamination. Indeed, despite lack of evidence of a new contamination source and with ongoing extensive flushing operations, oocysts remained detectable at low levels for up to 19 days after the change. Counts did generally decline during the 10 days after the supply was changed; however, counts peaked on March 20 after a burst in the main supply pipe. Increased counts on March 28–31 occurred when water samples started being taken from hydrants, rather than domestic taps. Hydrant water is discharged much more forcefully than that from domestic taps. The slow decline in oocyst counts after the change in supply may have been because of captured oocysts being released from the biofilm on the surface of the distribution pipes. Subsequent peaks associated with the burst and use of hydrants for sampling could have increased oocyst counts by stripping biofilm from the inner surface. *Cryptosporidium* oocysts do attach to biofilm in this manner (1,11,12)

Whatever the reasons for the continued detection of oocysts in water samples, few, if any, cases of infection were acquired after the source was changed. The epidemiologic analysis suggests that changing the water supply was the key public health measure. The boil-water advisory had little, if any, effect on reducing subsequent cases. The decision not to reintroduce the advisory when hydrant samples continued to show oocysts appears to have been justified.

Monitoring water samples, particularly with 10-L small-volume samples, highlighted the difficulties in interpreting the public health importance of oocysts in the water (13–15). Currently, the level of detectable *Cryptosporidium* oocysts in domestic water samples that poses no public health risk is unknown. The number of oocysts detected in the large-volume filtration of water from the WTW was below the limit currently defined as a national maximum permissible treatment standard (100 oocysts per 1,000 L) (2). However, this outbreak occurred 10 days after the most recent of three major rainfalls that could plausibly have given rise to contamination of the source water. Physical and computational fluid dynamics modeling suggested that the concentrations of oocysts in water leaving the WTW immediately after the heavy rainfall were 30 times the statutory treatment standard.

The introduction of continuous monitoring in the United Kingdom, together with existing surveillance for cryptosporidium infection in humans, will hopefully result in a better definition of an appropriate public health standard for this organism. However, recent human studies have shown a substantial intraspecies variability in the infectivity of *Cryptosporidium* oocysts (16). Furthermore, we have recently identified a novel strain of *C. parvum* that appears to be widespread in sheep but has never been described in humans (17). These observations suggest that identifying a standard in drinking water that would lead to a tolerable level of illness in the community may not be possible. Indeed, outbreaks of cryptosporidiosis associated with drinking water elsewhere in the United Kingdom have occurred despite the peak oocyst count’s being well within the statutory standard (18,19). Several episodes have also been reported in which high oocyst counts (>10 oocysts in 100 L) have been detected in treated water with no episodes of illness subsequently being detected in the community (20).

Further research is required to define the public health importance of low levels of *Cryptosporidium* oocysts as well as the optimal water sampling strategy during an outbreak. Similarly, the effectiveness and utility of system flushing remain to be shown. The current treatment standard should be reviewed, as further evidence relating to the public health impact of levels of *Cryptosporidium* oocysts becomes available.
